# Carcinoma of Unknown Original Identified as Renal Cell Carcinoma by 18F-Fluorodeoxyglucose Positron Emission Tomography/Computed Tomography Scan: A Report of Two Cases

**DOI:** 10.7759/cureus.29827

**Published:** 2022-10-02

**Authors:** Yumiko Kono, Keita Utsunomiya, Chisato Ohe, Nae Takizawa, Noboru Tanigawa

**Affiliations:** 1 Department of Radiology, Kansai Medical University, Osaka, JPN; 2 Department of Pathology and Laboratory Medicine, Kansai Medical University, Osaka, JPN; 3 Department of Urology and Andrology, Kansai Medical University, Osaka, JPN

**Keywords:** tumor imaging, renal cell metastasis, 18f-fluorodeoxyglucose positron emission tomography (18f-fdg pet), renal cell carcinoma, carcinoma of unknown origin

## Abstract

Imaging is useful in identifying the primary site of an unknown primary cancer, and ^18^F-fluorodeoxyglucose positron emission tomography/computed tomography (^18^F-FDG PET/CT) is an excellent imaging modality for identifying the primary lesion. However, a potential limitation is that ^18^F-FDG is physiologically excreted from the kidneys, thus masking renal lesions. In this report, we describe two cases of cancer of unknown origin that were detected as originating from renal cancer on ^18^F-FDG PET/CT. Both cases showed abnormal nodular accumulation of ^18^F-FDG in the kidney, which can be distinguished from the physiological excretion of ^18^F-FDG in the urinary tract. It is clinically crucial to be able to confirm the possibility of renal cancer, and careful observation of the urinary tract with ^18^F-FDG PET/CT can be useful.

## Introduction

Imaging helps locate the primary site of unknown primary cancer. ^18^F-fluorodeoxyglucose positron emission tomography/computed tomography (^18^F-FDG PET/CT) is an excellent imaging method for estimating the primary site of disease based on the distribution of lesions because it observes the increased glucose metabolism of the tumor throughout the body [1].

However, it has the potential limitation that renal lesions are masked due to the physiological excretion of ^18^F-FDG from the kidney. Clinical observations have shown unfavorable results for the role of ^18^F-FDG PET/CT in detecting and characterizing renal lesions, with a reported sensitivity of 46.6-62% in renal cell carcinoma [2-4].

On the other hand, Kang et al. reported that ^18^F-FDG PET/CT has the potential to predict the degree of differentiation of renal cancer and predict disease prognosis [3]. Some kidney cancers, e.g., sarcomatoid renal cell carcinoma, are small but can metastasize to multiple organs. In addition, these cancers require different treatment from other epithelial tumors, and the clinical value of diagnosis is high.

In this report, we describe two cases of small-size renal cancer identified by ^18^F-FDG PET/CT.

## Case presentation

**Figure 1:**
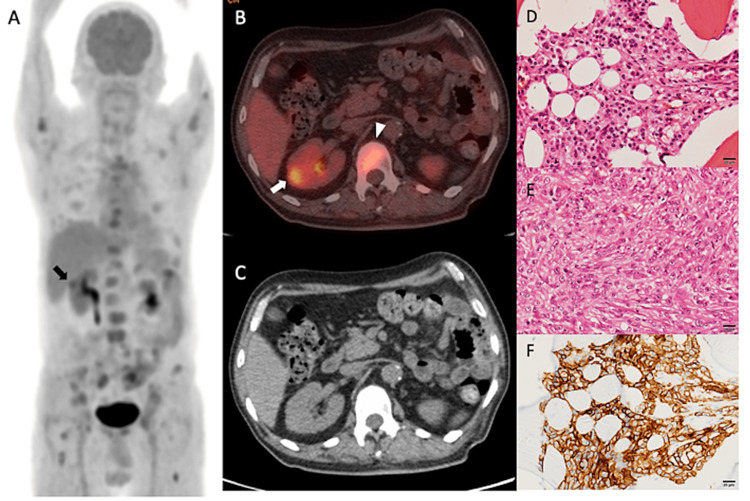
FDG PET/CT images of patient 1. (A) MIP; (B) axial view of ^18^F-FDG PET/CT fusion; (C) axial view of CT; (D) H&E staining in bone tumors, clear-cell renal cell carcinoma; (E) H&E staining, sarcomatoid features; (F) immunohistochemical staining, CA9; arrows: renal tumor; arrowhead: bone metastatic lesion. PET MIP imaging showed numerous abnormal accumulations in the bone and nodular accumulations in the right renal cortex (A and B), while CT showed no abnormalities in these areas (C). Histopathology of the bone biopsy showed CA-9-positive clear-cell renal cell carcinoma with sarcomatoid features. MIP: maximal intensity projection; CA-9: cancer antigen 9; ^18^F-FDG PET/CT: ^18^F-fluorodeoxyglucose positron emission tomography/computed tomography; H&E: hematoxylin and eosin

Patient 1 was a 72-year-old man with a family history of left renal stone, type 2 diabetes mellitus, old cerebral infarction, and old myocardial infarction. He had been fatigued for the past six months. His prior doctor suspected multiple bone metastases on magnetic resonance imaging (MRI), and bone biopsy revealed a metastatic bone tumor of unknown origin. A CT scan was performed, which failed to identify any renal lesions in the kidney. An ^18^F-FDG PET/CT was performed to detect primary carcinoma (Figure 1). There were many abnormal accumulations in the bone on FDG PET, and osteosclerosis was observed in some of these foci on CT. Interosseous replacement bone metastasis was diagnosed. The right kidney showed approximately 1.5 cm, localized nodular accumulation in the renal cortex. Bone biopsy specimens were immunostained for renal carcinoma and were positive for cancer antigen 9 (CA-9) and vimentin. The patient was diagnosed with clear-cell renal cell carcinoma with sarcomatoid features. A regimen of nivolumab 240 mg and ipilimumab 1 mg/kg intravenously every three weeks was initiated. After two courses, a progressive drug rash appeared. The patient died of toxic epidermal necrolysis and sepsis three months after the start of treatment.

**Figure 2:**
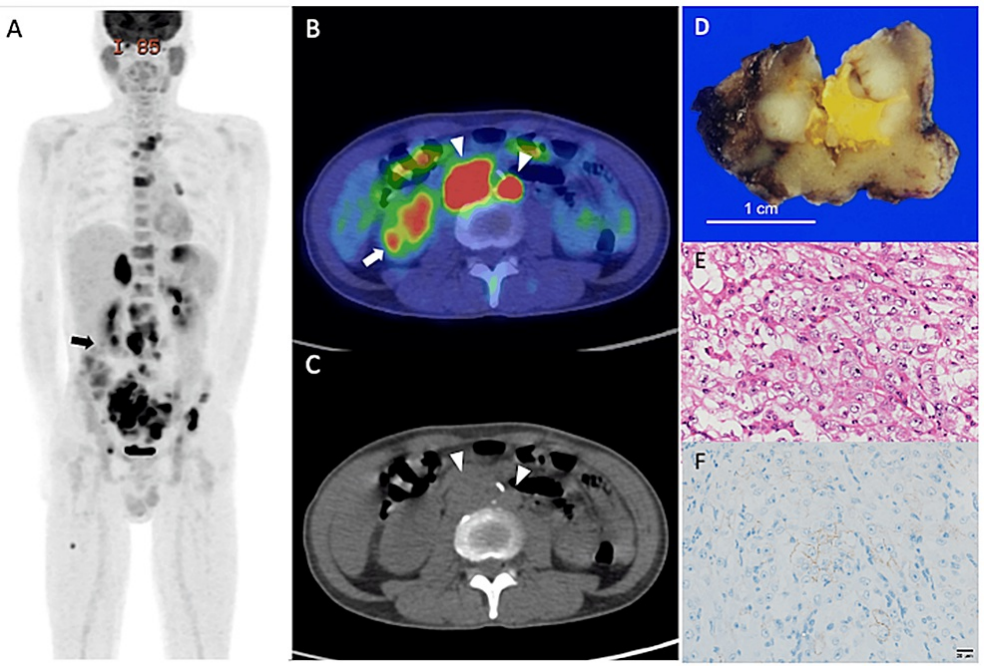
FDG PET/CT images of patient 2. (A) MIP; (B) axial view of ^18^F-FDG PET/CT fusion; (C) axial view of CT; (D) macroscopic image of a tumor removed in partial nephrectomy; (E) H&E staining; (F) Immunohistochemical staining, CA-9; arrows: renal tumor; arrowheads: lymph node metastasis. PET MIP image showed numerous abnormal accumulations in abdominal lymph nodes, left hilar lymph node, right adrenal gland, and multiple bones, as well as a punctate accumulation in the cortex of the right kidney separated from the urinary tract (A and B arrows). By contrast, no renal abnormalities could be noted on the CT scan (C). The resected renal tumor was diagnosed as immunohistochemically unclassified RCC (D-F). MIP: maximal intensity projection; CA-9: cancer antigen 9; ^18^F-FDG PET/CT: 18F-fluorodeoxyglucose positron emission tomography/computed tomography; H&E: hematoxylin and eosin; RCC: renal cell carcinoma

Patient 2 was a 47-year-old man with low back pain and right lower leg pain persisting for six months. A CT scan was performed, which identified multiregional enlargement of lymph nodes and right adrenal enlargement, although no renal lesions were detected. ^18^F-FDG PET/CT was performed to detect carcinoma, which showed multiple bone metastases with predominance in the pelvic bones (Figure 2). Abnormal accumulation of FDG was observed in the lymph nodes in the pararenal aorta and right iliac region and a 1.2 cm nodule in the left hilar pulmonary region. Increased accumulation of FDG was observed in the liver, right adrenal gland, and stomach. Esophagogastroduodenoscopy did not show a mass in the stomach. Lung cancer was suspected with multiple bones, liver, and right adrenal metastases. A right adrenal tumor biopsy was performed, and malignancy was diagnosed. However, immunostaining, which is highly specific for lung cancer, was negative, indicating that it was not lung cancer. Various immunostains revealed positivity for paired-box gene 8 (PAX8) and CA-9, and renal cell carcinoma was suspected. ^18^F-FDG PET/CT was re-evaluated, showing an approximately 8 mm spot accumulation in the right kidney. Subsequent diagnosis revealed a partial nephrectomy and a macroscopic 1.2 cm ill-defined nodular mass was revealed. The cut surface of the tumor was grayish-white with yellow foci. Histologically, the neoplastic cells with clear or eosinophilic cytoplasm showed a predominantly nested growth pattern. Immunohistochemically, tumor cells were positive for AE1/AE3, vimentin, PAX8, fumarate hydratase (FH) (retained), and integrase interactor 1 (INI1) (retained) but negative for CA-9, CD10, cathepsin K, and transcription factor binding to IGHM enhancer 3 (TFE3). Based on microscopic and immunohistochemical examinations, this case was diagnosed as unclassified renal cell carcinoma, staged pT1aN1MX1. A regimen of nivolumab 240 mg and ipilimumab 1 mg/kg intravenously every three weeks was initiated. After one year and three months, due to disease progression, the patient was switched to palliative care and transferred to a hospital.

## Discussion

The ^18^F-FDG PET/CT detection rate for cancer of unknown primary (CUP) has been reported to be 35.8-66.6% [5,6]. Although the detection rate of ^18^F-FDG PET/CT is relatively high in head and neck lesions, the reported detection rate for renal cancer is low, with a meta-analysis reporting a detection rate of less than 1.2% [1]. Greco et al. reported 24 cases (4.4%) of renal cell carcinoma diagnosed by immunohistology and genetic testing in 539 patients with CUP, suggesting a high false-negative rate on ^18^F-FDG PET/CT [7].

Because FDG is renally excreted physiologically, the diagnosis of the urinary tract in ^18^F-FDG PET/CT is difficult. Furthermore, because most renal cancers have weak FDG uptake, ^18^F-FDG PET/CT has a low sensitivity of 46.6-62% in detecting renal cell carcinoma, which is recommended for detecting recurrence and distant metastases [2-4]. Therefore, diagnosticians may lack attention to the urinary tract when searching for the primary tumor.

Although low-grade (G1, G2) renal carcinoma has a similar accumulation of FDG as in normal kidneys, high-grade (G3, G4) renal carcinoma, papillary renal cell carcinoma, and sarcomatoid degeneration have a solid accumulation [8]. Sarcomatoid dedifferentiation is found in about 4% of all renal cell carcinomas and about 20% of metastatic renal cell carcinomas [9,10]. Patients with sarcomatoid dedifferentiated renal cell carcinoma often present with progressive or metastatic disease and have a poor prognosis. According to the World Health Organization (WHO) guidelines, renal cell carcinoma with sarcomatoid dedifferentiation is considered a WHO-International Society of Urological Pathology grade 4. In both cases, the tumors were small, whereas FDG accumulation was observed, and distant metastasis was frequent, which was consistent with the conversion of a poorly differentiated sarcomatoid dedifferentiated-type renal cell carcinoma. Thus, with careful observation, PET/CT can adequately detect the primary site of renal cancer.

For the detection of renal cancer, the ultrasound has a high sensitivity of 82-83% and a specificity of 98-99% [11]. In contrast, systemic evaluation of the whole body is desirable first for searching for the primary site of CUP. On the other hand, in plain CT, where whole-body evaluation is possible, O'Connor et al. reported that the detection rate of renal cancer is as low as 37% for lesions less than 3 cm in diameter [12]. In both cases we experienced, the kidney cancer was less than 3 cm in diameter, and the CT we performed was unable to detect kidney cancer.

As carcinoma of unknown origin includes various advanced cancers, the prognosis is poor if an appropriate diagnosis is not made [13]. On the other hand, effective therapy for renal carcinoma has improved markedly in recent years with several molecularly targeted therapies and immune-checkpoint inhibitors [14]. In other words, the identification of renal cancer is of high clinical significance.

Besides, patients with occult renal cell carcinoma can be identified in the CUP group using molecular cancer classifier assay or specific renal immunohistochemistry stains [7]. In the two cases reported here, the renal lesions were detected by focusing on the urinary tract in consultation with our radiologist, pathologist, and urologic surgeon. An early and appropriate renal biopsy was performed to make a definitive diagnosis, leading to renal cell carcinoma treatment. Immunohistology can diagnose kidney cancer with high specificity. It is clinically essential to inform the pathologist of the possibility of kidney cancer and equally important to feed the immunohistological diagnosis back to the imaging diagnosis.

## Conclusions

Renal cancer may be missed on ^18^F-FDG PET/CT imaging to search for the primary site of CUP due to the bias that urinary tract lesions cannot be observed. In the case of a CUP, It is clinically crucial to be able to confirm the possibility of renal cancer, and careful observation of the urinary tract with ^18^F-FDG PET/CT can be useful.
